# Dioscin Protects ANIT-Induced Intrahepatic Cholestasis Through Regulating Transporters, Apoptosis and Oxidative Stress

**DOI:** 10.3389/fphar.2017.00116

**Published:** 2017-03-08

**Authors:** Hong Yao, Youwei Xu, Lianhong Yin, Xufeng Tao, Lina Xu, Yan Qi, Xu Han, Pengyuan Sun, Kexin Liu, Jinyong Peng

**Affiliations:** College of Pharmacy, Dalian Medical UniversityDalian, China

**Keywords:** dioscin, α-naphthylisothiocyanate, cholestasis, oxidative stress, PI3K/Akt pathway

## Abstract

Intrahepatic cholestasis, a clinical syndrome, is caused by excessive accumulation of bile acids in body and liver. Proper regulation of bile acids in liver cells is critical for liver injury. We previously reported the effects of dioscin against α-naphthylisothio- cyanate (ANIT)-induced cholestasis in rats. However, the pharmacological and mechanism data are limited. In our work, the animals of rats and mice, and Sandwich-cultured hepatocytes (SCHs) were caused by ANIT, and dioscin was used for the treatment. The results showed that dioscin markedly altered relative liver weights, restored ALT, AST, ALP, TBIL, GSH, GSH-Px, MDA, SOD levels, and rehabilitated ROS level and cell apoptosis. In mechanism study, dioscin not only significantly regulated the protein levels of Ntcp, OAT1, OCT1, Bsep and Mrp2 to accelerate bile acids excretion, but also regulated the expression levels of Bak, Bcl-xl, Bcl-2, Bax, Caspase 3 and Caspase 9 *in vivo* and *in vitro* to improve apoptosis. In addition, dioscin markedly inhibited PI3K/Akt pathway and up-regulated the levels of Nrf2, GCLc, GCLm, NQO1 and HO-1 against oxidative stress (OS) caused by bile acids. These results were further validated by inhibition of PI3K and Akt using the inhibitors of wortmannin and perifosine in SCHs. Our data showed that dioscin had good action against ANIT-caused intrahepatic cholestasis through regulating transporters, apoptosis and OS. This natural product can be considered as one active compound to treat intrahepatic cholestasis in the future.

## Introduction

Intrahepatic cholestasis is one common and acquired liver disease (Fuentes-Broto et al., 2009; Anwer, 2014). Current studies have focused on bile acid transporter dysregulation, oxidative stress (OS), hepatocyte apoptosis and inflammation associated with cholestasis (Roma and Sanchez Pozzi, 2008; Gonzalez-Sanchez et al., 2015). Cholestasis is defined as the impairment of bile and bilirubin secretion (Schmitt et al., 2000), and which the accumulation of bile salts can affect the functions and expression levels of some transporters in hepatocytes (Akita et al., 2001; Kawai et al., 2007). Bile acid and bilirubin can be excreted from liver via Na^+^-taurocholate co-transporting polypeptide (Ntcp) and Bsep (Meier and Stieger, 2002; Arrese and Trauner, 2003). Bilirubin is produced by the hepatic organic anion transporting polypeptides (OATPs) and organic cation transporters (OCTs), which is then secreted into the tubules through multidrug resistance associated protein 2 (Mrp2) (Zollner et al., 2001; Kalliokoski and Niemi, 2009; Saeki et al., 2011). Thus, regulating the expression levels and functions of these membrane transporters might improve cholestasis.

The abnormal metabolism of bile acids can cause apoptosis (Woolbright et al., 2013). Apoptosis refers to the stable and orderly death of cells which are controlled by genes in order to maintain the stability of the internal environment (Perez et al., 2005). Apoptosis is a process controlled by multiple genes including apoptosis- related cysteine protease 9 (Caspase 9) and Caspase 3 (Yerushalmi et al., 2001).

Over-production of ROS can cause cell damage via regulating signal transduction pathways associated with oxidation (Cesaratto et al., 2004). Recently, Nrf2 shows active effect against OS, and Nrf2 translocation can ultimately inhibit the system by regulating GSH positive regulatory signals, GCLc and GCLm (Okada et al., 2009; Wang G. et al., 2014). Once the GCLc and GCLm increase the intracellular concentration of GSH, the reduction of ROS production has been provided for protecting the operating system (Xu et al., 2008). The PI3K/Akt pathway is one potential upstream signal transduction regulator for Nrf2. Accumulating evidence suggests that Nrf2 nuclear translocation and GSH synthesis may be mediated by phosphorylation of Akt (Li et al., 2014). Thus, activation of glutathione and PI3K/ Akt signaling pathway play important roles in cholestasis.

Chinese medicines have shown their unique roles in the treatment of cholestasis, and Chinese medicines are rich sources of bioactive substances that can be used to prevent human disease (Balunas and Kinghorn, 2005). Currently, many herbal extracts and natural products with protective actions against cholestasis have been reported (Chen et al., 2016).

Dioscin (Dio, Supplementary Figure 1) widely exists in some herbs (Yin et al., 2010). Pharmacological studies have demonstrated that this natural product shows active effects against cancer (Hsieh et al., 2013), hyperlipidemic (Li et al., 2010) and fungus (Li et al., 2003). In previous works, we found that dioscin had good effects against liver fibrosis (Liu et al., 2015; Zhang et al., 2015), acute liver damages (Lu et al., 2012; Yao et al., 2016), obesity (Liu et al., 2015), osteoporosis (Tao et al., 2016), renal and hepatic ischemia/reperfusion injury (Tao et al., 2014; Qi et al., 2015). Importantly, dioscin has active effect against α- naphthylisothiocyanate (ANIT)-induced liver cholestasis (Zhang et al., 2016). However, the reported data are limited to show the comprehensive actions and mechanisms of dioscin against ANIT-induced liver cholestasis.

Therefore, in the present study, we aimed to explore on reducing ANIT-induced intrahepatic cholestasis in the role and mechanism of dioscin.

## Materials and Methods

### Chemicals

Dioscin (>98%) was prepared from *Dioscorea nipponica* Makino in our laboratory (Yin et al., 2010), which was dissolved with 0.1% dimethylsulfoxide (DMSO) for *in vitro* experiments, or with 0.5% carboxymethylcellulose sodium (CMC-Na) solution for *in vivo* tests. CMC-Na, Tris, Sodium dodecyl sulfate (SDS) and 4′,6′-diamidino-2-phenylindole (DAPI) were purchased from Sigma (St. Louis, MO, USA). ALT, AST kits, ALP, TBIL, GSH, GSH-Px, MDA, and SOD were obtained from the Nanjing Jiancheng Institute of Biotechnology (Nanjing, China). A tissue protein extraction kit was obtained from Keygen Biotech. Co., Ltd. (Nanjing, China). *In Situ* Cell Death Detection Kit (TMR Red; Roche, NJ, USA). RNAiso Plus, a PrimeScript^®^ RT Reagent Kit with gDNA Eraser (Perfect Real Time) and SYBR^®^ Premix Ex Taq^TM^ II (Tli RNase H Plus) were purchased from TaKaRa Biotechnology Co., Ltd. (Dalian, China). A bicinchoninic acid (BCA) protein assay kit was purchased from the Beyotime Institute of Biotechnology (Jiangsu, China).

### Cell Isolation and Culture

Sandwich-cultured hepatocytes (SCHs) were isolated from male Wistar rats (200 ± 20 g) by two-step perfusion and purified by 45% isotonic Percoll (Kotani et al., 2011). As shown in Supplementary Figure 2, the hepatocytes were seeded onto collagen -coated plates. Twenty-four hours after seeding, the hepatocytes were overlaid with 0.25 mg/mL Matrigel Basement Membrane Matrix (BD Biosciences, San Jose, CA, USA) to form sandwich configuration and cultured as described previously (Chandra et al., 2005; Yan et al., 2011). After 24 h, the SCHs were exposed to 100 μM of ANIT (dissolved in DMSO, 0.1% final concentration).

### Cell Toxicity Assay

The primary cultured hepatocytes were plated into 96-well plates at a density of 5 × 10^4^ cells/mL (100 μL) per well overnight in an incubator and incubated until they reached to approximately 70% confluence. Next, the cells were pretreated with various concentrations of dioscin (50, 100, 200, 400, 800, 1600, and 3200 ng/mL) for 24 h at 37°C, and then the toxicity of the compound was assayed using the MTT method.

### Cell Proliferation Assay

The primary cultured hepatocytes were seeded in 96-well plates at a density of 5 × 10^4^ cells/mL (100 μL) for 24 h before treatment. According to the cell proliferation assay kit to add Brdu. After 6 h, the cells were then treated with dioscin at the concentrations of 200, 400, and 800 ng/mL for 6, 12, and 24 h at 37°C, and the cells proliferation were measured using the Brdu method.

### Detection of Intracellular ROS Level

The SCHs were plated in 6-well plates at a density of 5 × 10^4^ cells/mL (1 mL) and treated with dioscin at the concentrations of 200, 400, and 800 ng/mL for 24 h, then exposed to 100 μM of ANIT for 24 h. The cells were harvested and re-suspended in 1 mL dichlorodihydrofluorescein diacetate (DCFH-DA) (10 μM) for the detection of ROS level, which was imaged by fluorescence microscope (Olympus, Tokyo, Japan).

### Detection of Apoptosis

Terminal deoxynucleotidyl transferase mediated dUTP nick end labeling (TUNEL) staining and *in situ* cell apoptosis detection kit (TMR red; Roche, NJ, USA) according to manufacturer’s instructions. Images are taken with a fluorescence microscope (OLYMPUS, Tokyo, Japan).

### ANIT-Induced Intrahepatic Cholestasis

Male Wistar rats (200 ± 20 g) and male C57BL/6J mice (20 ± 2 g) were purchased from the Experimental Animal Centre of Dalian Medical University, Dalian, China (quality certificate number: SCXK (Liao) 2013-0003). After 1 week of acclimatization, the animals were randomly divided into five groups (*n* = 10): control group, model (ANIT) group, and high, middle and lose-dose of dioscin-treated groups. The animals in dioscin-treated groups were administered with dioscin at the doses of 60, 30, and 15 mg/kg for rats, and 80, 40, and 20 mg/kg for mice for 7 consecutive days. Liver injury was induced by intraperitoneal (i.p.) ANIT (60 mg/kg for rats and 80 mg/kg for mice) 2 h before the last administration. After 7 days, the animals were sacrificed after an overnight fast. Then, the blood and liver tissue were collected and stored for further analysis. In tests, all animals were housed in a controlled environment at 23 ± 2°C for a period of a 12-h dark/light free access to food and water. All the experimental procedures were approved by the animal protection and use Committee of Dalian Medical University and strictly in accordance with the laws of People’s Republic of China Legislation Regarding the Use and Care of Laboratory Animals.

### Serum Biochemistry Assay

The serum levels of ALT, AST ALP and TBIL were determined using the commercial kits according to the manufacturer’s instructions.

### Antioxidant Assay

The levels of GSH, GSH-Px, MDA and SOD in liver tissues were measured according to the manufacturer’s instructions.

### Histological and Immunohistochemical Assays

The liver tissues were fixed in 10% formalin and embedded in paraffin. Five-micron -thick sections were stained with haematoxylin-eosin (H&E). Images were acquired by light microscopy (Nikon Eclipse TE2000-U, Nikon, Japan), and the degree of liver injury was quantified using Image-Pro Plus 6.0 software. Immunofluorescence staining of tissue slices or formal in-fixed cells for Ntcp and p-PI3K were preformed using primary antibodies, respectively (Santa Cruz, CA, USA) in a humidified chamber at 4°C overnight. After washing twice in PBS, the cells and liver tissue sections were incubated with a fluorescein-labeled secondary antibody for 1 h. Eventually, the cell nuclei were stained with DAPI (5 μg/mL). All samples were imaged using a fluorescence microscope (Olympus, Tokyo, Japan).

### Quantitative Real-Time PCR Assay

Total RNA samples from the cells and livers were extracted using RNAiso Plus reagent following the manufacturer’s protocol. Reverse transcription for cDNA synthesis and quantitative real-time PCR were performed as previously described. The forward (F) and reverse (R) primers for the tested genes are listed in Supplementary Table 1. For each sample, the *Ct* values for the target gene and GAPDH (as a calibrator) were determined based on standard curves. The calculated relative *Ct* value of each gene was divided by the relative value of GAPDH. Then, the expression level of each gene in the control group was set to one-fold and used to determine the relative levels in the other samples (*n*-fold).

### Western Blotting Assay

The protein samples from the cells and liver tissues were extracted following standard protocols (Beyotime Biotechnology, Haimen, China), and the protein content was determined using a BCA Protein Assay Kit. Proteins were subjected to SDS-PAGE and then transferred to a nitrocellulose membrane. After blocking, the membranes were incubated for 1 h at room temperature or overnight at 4°C. The primary antibodies are listed in Supplementary Table 2. The blots were then incubated with horseradish peroxidise-conjugated antibodies for 2 h at room temperature at a 1: 2000 dilution (Beyotime Institute of Biotechnology, China). Protein expression was detected by the enhanced chemiluminescence (ECL) method and imaged with a Bio-Spectrum Gel Imaging System (UVP, USA). To eliminate variations due to protein quantity and quality, the data were adjusted to GAPDH expression (IOD of objective protein versus IOD of GAPDH protein).

### Inhibition of PI3K and Akt in SCHs

Sandwich-cultured hepatocytes were plated into 6-well plates (5 × 10^4^ cells/mL), and then exposed to PI3K inhibitor wortmannin and Akt inhibitor perifosine (Beyotime, Jiangsu, China) for 2 h. After incubation, the cells were pre-treated with dioscin (800 ng/mL) and then treated with ANIT (100 μM) for 24 h. At last, the protein levels of p-PI3K, p-Akt, Nrf2, GCLm, GCLc, NQO1, HO-1, and the mRNA levels of Nrf2, GCLm, GCLc, NQO1, HO-1 were determined.

### Statistical Analyses

Data were evaluated as the mean ± standard deviation (mean ± SD). Statistical analysis of the quantitative data for multiple group comparisons was performed using one-way analysis of variance (ANOVA) followed by Duncan’s test, whereas paired comparisons were performed using the *t*-test with SPSS software (ver. 20.0; SPSS, Chicago, IL, USA). The results were considered to be significant at ^∗^*p* < 0.05 or ^∗∗^*p* < 0.01.

## Results

### Effect of Dioscin on the Proliferation of Primary Cultured Hepatocytes

As shown in Supplementary Figures 3A,B, dioscin (50, 100, 200, 400, and 800 ng/mL) for the primary cultured hepatocytes under 24 h treatment had no effect on cell viability. Dioscin (200, 400, and 800 ng/mL) for 6, 12, and 24 h significantly changed cell proliferation compared with control group. Therefore, dioscin at the concentrations of 200, 400 and 800 ng/mL under 24 h was selected to protect ANIT-induced cholestasis.

### Dioscin Rehabilitates ANIT-Induced Cholestasis *In vivo*

**FIGURE 1:**
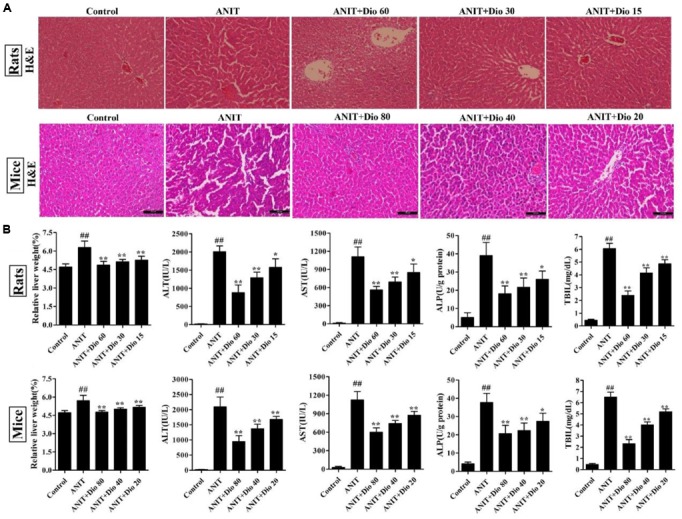
**Dioscin rehabilitates ANIT-induced cholestasis *in vivo*. (A)** Effects of dioscin on ANIT-induced liver injury based on H&E staining (× 200 original magnification). **(B)** Effects of dioscin on the relative liver weight and the serum levels of ALT, AST, ALP and TBIL in rats and mice. Values are expressed as the mean ± SD (*n* = 10). ^##^*p* < 0.01 compared with control groups. ^∗^*p* < 0.05 and ^∗∗^*p* < 0.01 compared with model groups.

As shown in **Figure 1A**, the livers in control animals showed normal architecture, and the damages including cell necrosis and inflammatory cell infiltration were observed in ANIT-treated animals, which were significantly rehabilitated by the compound. Furthermore, dioscin markedly restored the relative liver weight, serum AST, ALT, ALP and TBIL levels (**Figure 1B**).

### Dioscin Reduces ROS Level in SCHs

**FIGURE 2:**
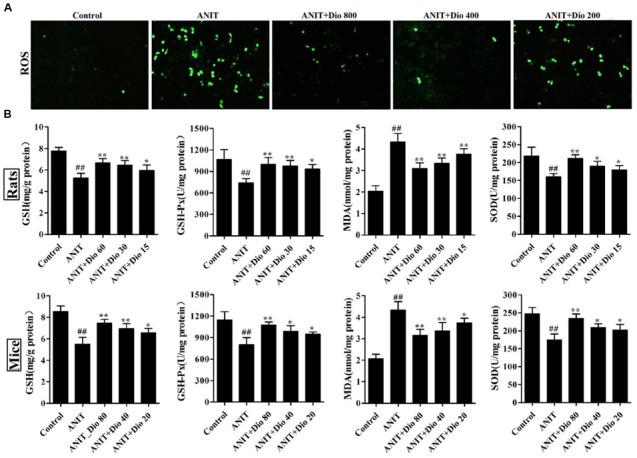
**Dioscin attenuates ANIT-induced oxidative stress. (A)** Effects of dioscin on the ROS levels in SCHs by immunofluorescence assay (× 400 magnification). **(B)** Effects of dioscin on the levels of GSH, GSH-Px, MDA and SOD in rats and mice. Values are expressed as the mean ± SD (*n* = 10). ^##^*p* < 0.01 compared with control groups. ^∗^*p* < 0.05 and ^∗∗^*p* < 0.01 compared with model groups.

As shown in **Figure 2A**, compared with control group, dioscin markedly decreased ROS level in SCHs.

### Dioscin Reduces Attenuates Oxidative Stress *In vivo*

As shown in **Figure 2B**, dioscin significantly reversed the levels of GSH, GSH-Px, SOD and MDA compared with ANIT model groups.

### Dioscin Regulates ANIT-Induced Cholestasis by Transporters

**FIGURE 3:**
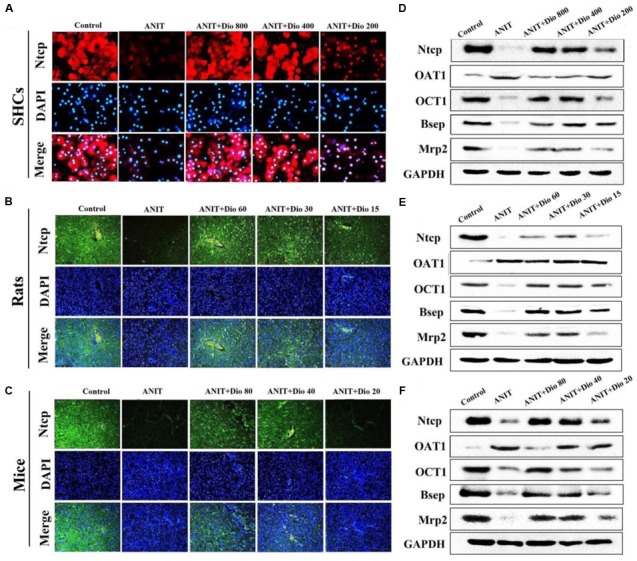
**Dioscin regulates ANIT-induced cholestasis by transporters. (A–C)** Effects of dioscin on the levels of Ntcp based on immunofluorescence assay in SCHs (× 400 original magnification) and animals (× 200 original magnification). **(D–F)** Effects of dioscin on the protein levels of Ntcp, OAT1, OCT1, Bsep and Mrp2 in SCHs, rats and mice.

As shown in **Figures 3A–C**, doscin clearly increased the expression levels of Ntcp *in vivo* and *in vitro* based on immunofluorescence assay. The protein levels of Ntcp, OCT1, Bsep and Mrp2 were markedly down-regulated, and the levels of OAT1 were obviously increased in model groups compared with normal groups, which were significantly restored by dioscin (**Figures 3D–F** and Supplementary Figures 4A–C). Together, these data indicated that dioscin improved cholestasis via regulating the transporters.

### Dioscin Reduces the Apoptosis Caused by ANIT

**FIGURE 4:**
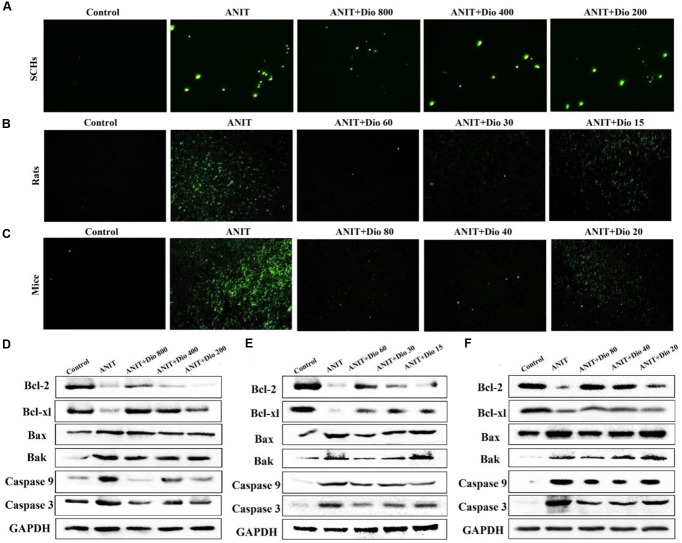
**Dioscin attenuates ANIT-induced apoptosis. (A–C)** Representative images of TUNEL-stained SCHs ( × 400 magnification), and the livers of rats and mice ( × 200 magnification). **(D–F)** Effects of dioscin on the protein levels of Bcl-2, Bcl-xl, Bax, Bak, Caspase 9 and Caspase 3 in SCHs, rats and mice.

As shown in **Figures 4A–C**, more positive cells with green fluorescence were found in model groups than those of in dioscin-treated groups in SCHs, rats and mice.

### Dioscin Regulates ANIT-Induced Apoptosis *In vivo*

As shown in **Figures 4D–F** and Supplementary Figures 5A–C, dioscin significantly regulated the protein levels of Bak, Bcl-xl, Bax, Bcl-2, Casepase 3 and Caspase 9 compared with ANIT model groups.

### Dioscin Regulates PI3K/Akt Signal Pathway

**FIGURE 5:**
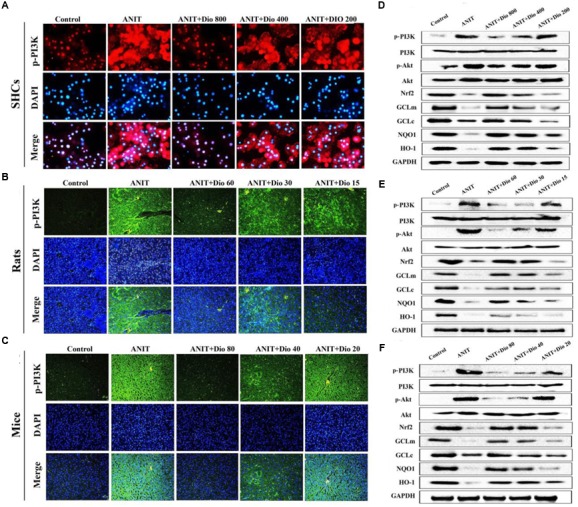
**Dioscin inhibits PI3K/Akt signaling pathway. (A–C)** Effects of dioscin on PI3K levels based on immunofluorescence assay in SCHs ( × 400 original magnification) and animals ( × 200 original magnification). **(D–F)** Effects of dioscin on the protein levels of pPI3K, p-Akt, Nrf2, GCLm, GCLc, NQO1 and HO-1 in SCHs, rats and mice.

Doscin obviously down-regulated p-PI3K levels based on immunofluorescence assay *in vitro* and *in vivo* (**Figures 5A–C**). Compared with ANIT groups, the protein levels of p-PI3K and p-Akt were significantly down- regulated by dioscin, while the levels of Nrf2, GCLm, GCLc, NQO1 and HO-1 were markedly up- regulated by dioscin (**Figures 5D–F** and Supplementary Figures 6A–C).

### Dioscin Inhibits PI3K/Akt Mediated-Oxidative Stress

**FIGURE 6:**
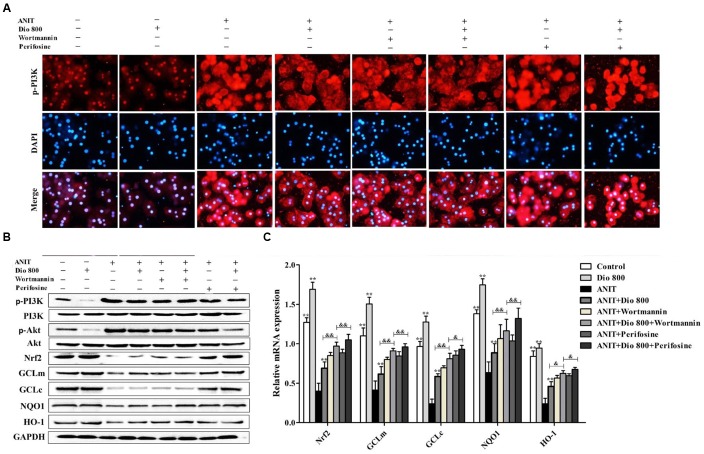
**Dioscin inhibits PI3K/Akt-mediated oxidative stress. (A)** Effects dioscin on p-PI3K levels based on immunofluorescence assay in SCHs ( × 400 original magnification) treated by wortmannin and perifosine. **(B)** Effects of dioscin on the protein levels of p-PI3K, p-Akt, Nrf2, GCLm, GCLc, NQO1 and HO-1 in SCHs treated by wortmannin and perifosine. **(C)** Effects of dioscin on the mRNA levels of Nrf2, GCLm, GCLc, NQO1 and HO-1 in SCHs treated by wortmannin or perifosine. Values are expressed as the mean ± SD (*n* = 5). ^∗^,^∗∗^*p* < 0.01 compared with model group; ^&^*p* < 0.05 and ^&&^*p* < 0.01 compared with ANIT + Dio 800 + wortmannin group.

As shown in **Figure 6A**, treatment of SCHs with wortmannin for 2 h significantly inhibited p-PI3K expression, whereas treatment with perifosine for 2 h showed no significant effect on p-PI3K level. In addition, the protein levels of pPI3K, p-Akt, Nrf2, GCLm, GCLc, NQO1 and HO-1 (**Figure 6B** and Supplementary Figure 7), and the mRNA levels of Nrf2, GCLm, GCLc, NQO1 and HO-1 (**Figure 6C**) were partially regulated by wortmannin. No obvious changes in p-PI3K levels were found in SCHs by perifosine. These data indicated that dioscin inhibited OS via PI3K/Akt pathway.

## Discussion

Accumulation of toxic bile acids in liver can cause cholestasis (Padda et al., 2011) with the main features of including primary sclerosing cholangitis, biliary cirrhosis and atresia (Cui et al., 2009; Weerachayaphorn et al., 2014). Accumulating event suggests that free radicals are associated with apoptosis and lipid peroxidation in cholestatic lesions (Yang et al., 2009), and we believe that OS is a key factor for cholestatic damage (Ma et al., 2014). ANIT, a commonly used hepatotoxicant, can interrupt bile flow and accumulate bile acids in liver (Ohta et al., 1999). The changes of antioxidant defense system and lipid peroxidation are the primary contributors to liver damage caused by ANIT (Tanaka et al., 2009; Wang T. et al., 2014). In the present work, dioscin markedly altered relative liver weights, and restored ALT, AST, ALP, TBIL levels, suggesting that dioscin may be one active compound to treat intrahepatic cholestasis.

Some exogenous and endogenous chemials are mediated by transporters. Basolateral transporters, including Ntcp, OCT1 and OAT1, can transport bile acids and organic anions, and canalicular transporters including Mrp2 and Bsep can transport chemicals from hepatocytes into bile (Tanaka et al., 2006, 2009). The base of the liver is transported to the bilirubin and bile acids in the liver and the liver cells enter the blood. Previous study has shown the protective action of dioscin against cholestasis caused by ANIT in rats via adjusting Bsep, Mrp2 and Oatps (Zhang et al., 2016). In this paper, dioscin not only significantly regulated the levels of OAT1, Mrp2, OCT1 and Bsep, but also regulated Ntcp levels in rats, mice and SCHs. Above all, the action of dioscin against cholestasis caused by ANIT was due to hepatic transporters.

Bile acids accumulated in livers can lead to OS and apoptosis (Zollner et al., 2003; Li et al., 2011). Apoptosis is a process controlled by multiple genes including Bcl-2 family, caspase family, cancer genes and tumor suppressor genes (Padda et al., 2011; Tao et al., 2014). In the present study, disocin significantly increased the levels of Bcl-xl, Bcl-2, and decreased the levels of Bak, Caspase 3,Bax and Caspase 9 *in vivo* and *in vitro* to reduce apoptosis, suggesting that suppression of apoptosis may be one potential mechanism of dioscin against ANIT-induced liver injury.

It has been suggested that PI3K/Akt pathway can cause a signal for the operating system by regulating Nrf2 expression. Even in hepatocytes, the activity of the GCL subunit is regulated by PI3K/Akt signal (Anwer, 2014). Akt, a major regulator of PI3K signal, shows anti-apoptotic effect that is phosphorylated and activated in many different forms of cell death (Arisawa et al., 2009). A key transcription factor, Nrf2 is a regulator of GCL expression in response to OS and other anti OS genes. Under normal circumstances, Nrf2 is a repressed cytoplasmic actin binding protein Keap1 (Lu, 2009; Wu et al., 2014). The reported paper has shown that the activation of Nrf2 can protect cholestasis via regulating several antioxidant enzymes (Yeh et al., 2015). Upon OS, Nrf2 can initiate the transcription of some genes including antioxidant enzymes (Zhao et al., 2013). Further researches have shown that Nrf2 can regulate glutamine synthetase and GCL subunits (Aleksunes et al., 2006; Oguz et al., 2012). In addition, two antioxidation elements, the prototypical Nrf2-targeted genes are HO-1 and NQO1 (Ko et al., 2008). In our paper, dioscin obviously reversed the levels of GSH-Px, GSH, MDA, SOD *in vivo*, and rehabilitated ROS level in cells. In addition, dioscin significantly adjusted the expression levels of Nrf2, GCLc, GCLm, NQO1 and HO-1, indicating the potent antioxidation effect of dioscin through Nrf2. We further investigated the actions of PI3K/Akt signal on controlling Nrf2 translocation and GSH level. All the evidences mentioned here indicated that dioscin can control cholestasis caused by ANIT to down-regulate OS through PI3K/Akt pathway.

## Conclusion

**FIGURE 7:**
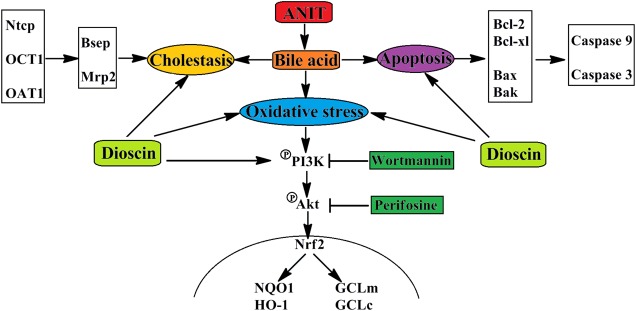
**The schematic diagram of dioscin against ANIT-induced intrahepatic cholestasis.** Dioscin regulated the levels of Ntcp, OAT1, OCT1, Bsep and Mrp2 to accelerate bile acids excretion, and then adjusted the levels of Bcl-2, Bcl-xl, Bax, Bak, Caspase 9 and Caspase 3 to alleviate apoptosis. In addition, dioscin markedly inhibited PI3K/Akt pathway and up-regulated the levels of Nrf2, GCLc, GCLm, NQO1 and HO-1 against oxidative stress caused by bile acids. Dioscin exhibited protective effect against ANIT-induced intrahepatic cholestasis via altering transporters, apoptosis and oxidative stress.

In conclusions, dioscin markedly attenuated cholestasis through regulating transporters, apoptosis and OS (**Figure 7**), which showed that this natural product can be considered as an anti-apoptosis and anti-oxidative compound to treat cholestasis in future.

## Author Contributions

HY was responsible for the planning, execution of all experiments and preparation of the manuscript. YX, LY, and XT were responsible for ANIT-induced intrahepatic cholestasis model experiments. LX, YQ, XH, PS, and KL were responsible for the preparation, isolation and bioavailability study of dioscin. JP provided critical inputs for the experiments. JP was responsible for the conceptualization, planning, execution and troubleshooting of the experiments, preparation of the manuscript and the financial support.

## Conflict of Interest Statement

The authors declare that the research was conducted in the absence of any commercial or financial relationships that could be construed as a potential conflict of interest.

## Abbreviations

Akt: protein kinase B
ALP: alkaline phosphatase
ALT: alanine aminotransferase
AST: aspartate aminotransferase
Bax: BCL2-associated X protein
Bak: BCL2-killer
Bcl-2: B-cell lymphoma-2
Bcl-xl: BCL2-like 1
Bsep: bile salt export pump
Caspase 3: apoptosis-related cysteine protease 3
Caspase 9: apoptosis-related cysteine protease 9
GCLc: glutathione cysteine ligase catalytic subunit
GCLm: glutathione cysteine ligase modifier subunit
GSH: glutathione
GSH-Px: glutathione peroxidase
HO-1: heme oxygenase-1
MDA: malondialdehyde
Mrp2: multidrug resistance protein 2
Nrf2: nuclear erythroid factor 2-related factor 2
Ntcp: Na^+^- taurocholate co-transporting polypeptide
NQO1: NADH dehydrogenase quinone 1
OAT1: organic anion transporter 1
OCT1: organic cation transporter 1
PI3K: phosphatidylinositol 3-kinase
ROS: reactive oxygen species
SOD: superoxide dismutase
TBIL: total bilirubin
