# Supplementary material for: Dioscin Protects ANIT-Induced Intrahepatic Cholestasis Through Regulating Transporters, Apoptosis and Oxidative Stress
Source: Front Pharmacol. 2017 Mar 8;8:116. doi: 10.3389/fphar.2017.00116 (PMC5340742; doi:10.3389/fphar.2017.00116)
Supplement: Supplementary file 1 [file Presentation_1.PDF]

Dioscin protects ANIT-induced intrahepatic cholestasis through regulating transporters, apoptosis and oxidative stress

Hong Yao, Youwei Xu, Lianhong Yin, Xufeng Tao, Lina Xu, Yan Qi, Xu Han, Pengyuan Sun, Kexin Liu, Jinyong Peng*

College of Pharmacy, Dalian Medical University, Western 9 Lvshunnan Road, Dalian 116044, China

Corresponding author,
Dr. Jinyong Peng
College of Pharmacy
Dalian Medical University
Dalian, China
Tel.: +86 411 8611 0411
Fax: +86 411 8611 0411
Email: jinyongpeng2008@126.com

Supplemental Table 1. The primer sequences used for real-time PCR assay in the present work.

Gene	Forward primer (5'-3')	Reverse primer (5'-3')
Rat GAPDH	GGCACAGTCAAGGCTGAGAATG	ATGGTGGTGAAGACGCCAGTA
Rat Nrf2	CAAACATTCAAGCCGATTAGAGG	CGGCAACTTTATTCTTCCCTCT
Rat GCLm	TGATGCCACCAGATTTGACTGC	CTGCTTTTCACGATGACCGAGT
Rat GCLc	TCTGGATGCCAACGAGTC	CCTGGAGCAGTACCACAAATA
Rat NQO1	GCGGTGAGAAGAGCCCTGAT	GCTCCCCTGTGATGTCGTTTC
Rat HO-1	CAGGGTGACAGAAGAGGCTAAG	CTGTGAGGGACTCTGGTCTTTG

Supplemental Table 2. The information of the antibodies used in the present work.

Antibody	Source	Dilutions	Company
GAPDH	Rabbit	1:2000	Proteintech Group, Chicago, USA
Bcl-2	Rabbit	1:1000	Proteintech Group, Chicago, USA
Bcl-xl	Rabbit	1:1000	Proteintech Group, Chicago, USA
Bax	Rabbit	1:1000	Proteintech Group, Chicago, USA
Bak	Rabbit	1:1000	Proteintech Group, Chicago, USA
Caspase 9	Rabbit	1:1000	Proteintech Group, Chicago, USA
Caspase 3	Rabbit	1:1000	Proteintech Group, Chicago, USA
Ntcp	Rabbit	1:2000	Santa Cruz, California, USA
OAT1	Rabbit	1:2000	Santa Cruz, California, USA
OCT1	Rabbit	1:1000	Proteintech Group, Chicago, USA
Bsep	Rabbit	1:2000	Santa Cruz, California, USA
Mrp2	Rabbit	1:1000	Proteintech Group, Chicago, USA
p-PI3K	Rabbit	1:2000	Santa Cruz, California, USA
PI3K	Rabbit	1:2000	Santa Cruz, California, USA
p-Akt	Rabbit	1:2000	Santa Cruz, California, USA
Akt	Rabbit	1:2000	Santa Cruz, California, USA
Nrf2	Rabbit	1:1000	Proteintech Group, Chicago, USA
GCLm	Rabbit	1:1000	Proteintech Group, Chicago, USA
GCLc	Rabbit	1:1000	Proteintech Group, Chicago, USA
NQO1	Rabbit	1:1000	Proteintech Group, Chicago, USA
HO-1	Rabbit	1:1000	Proteintech Group, Chicago, USA

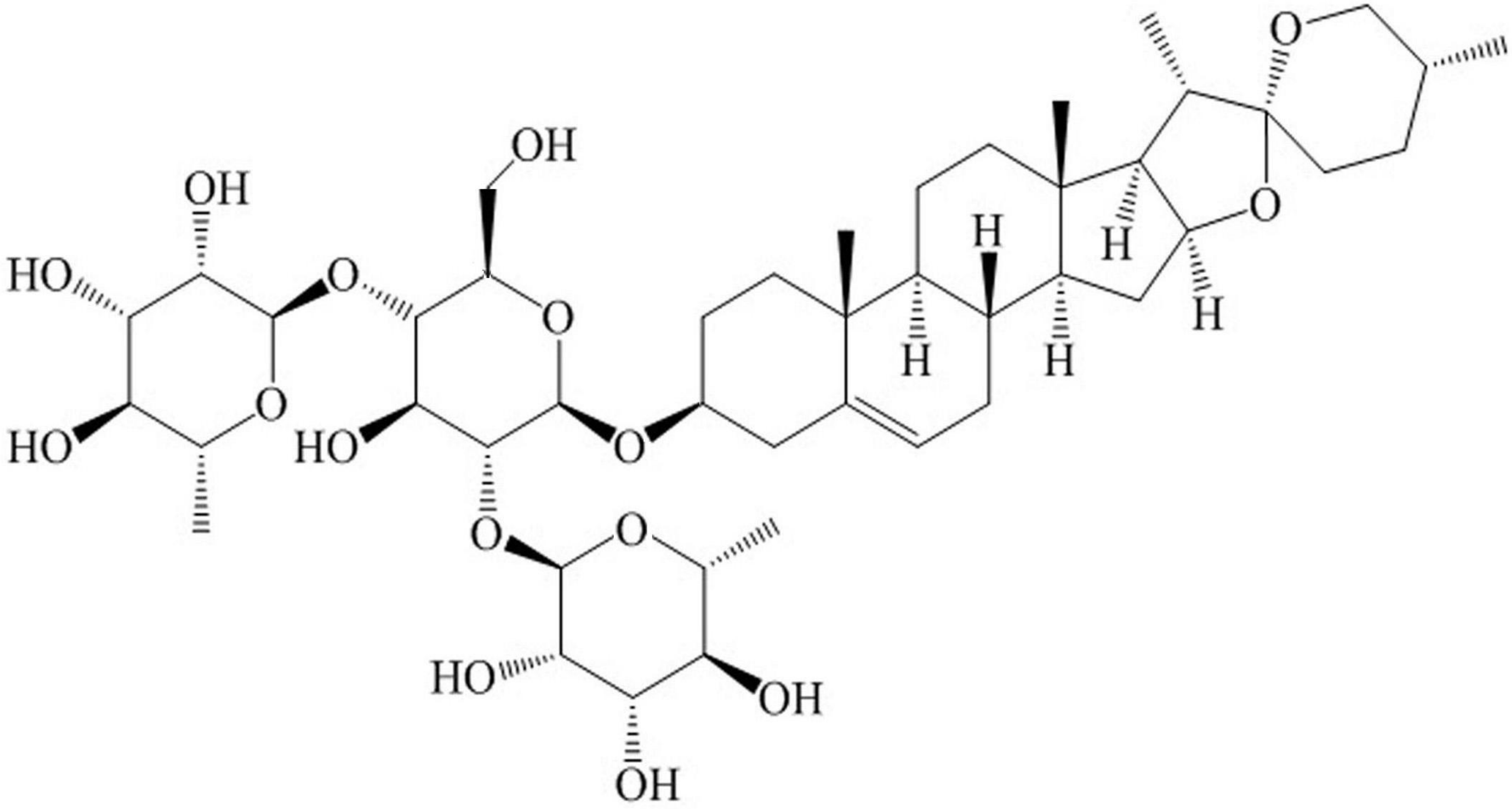

Supplemental Figure 1. The chemical structure of dioscin.

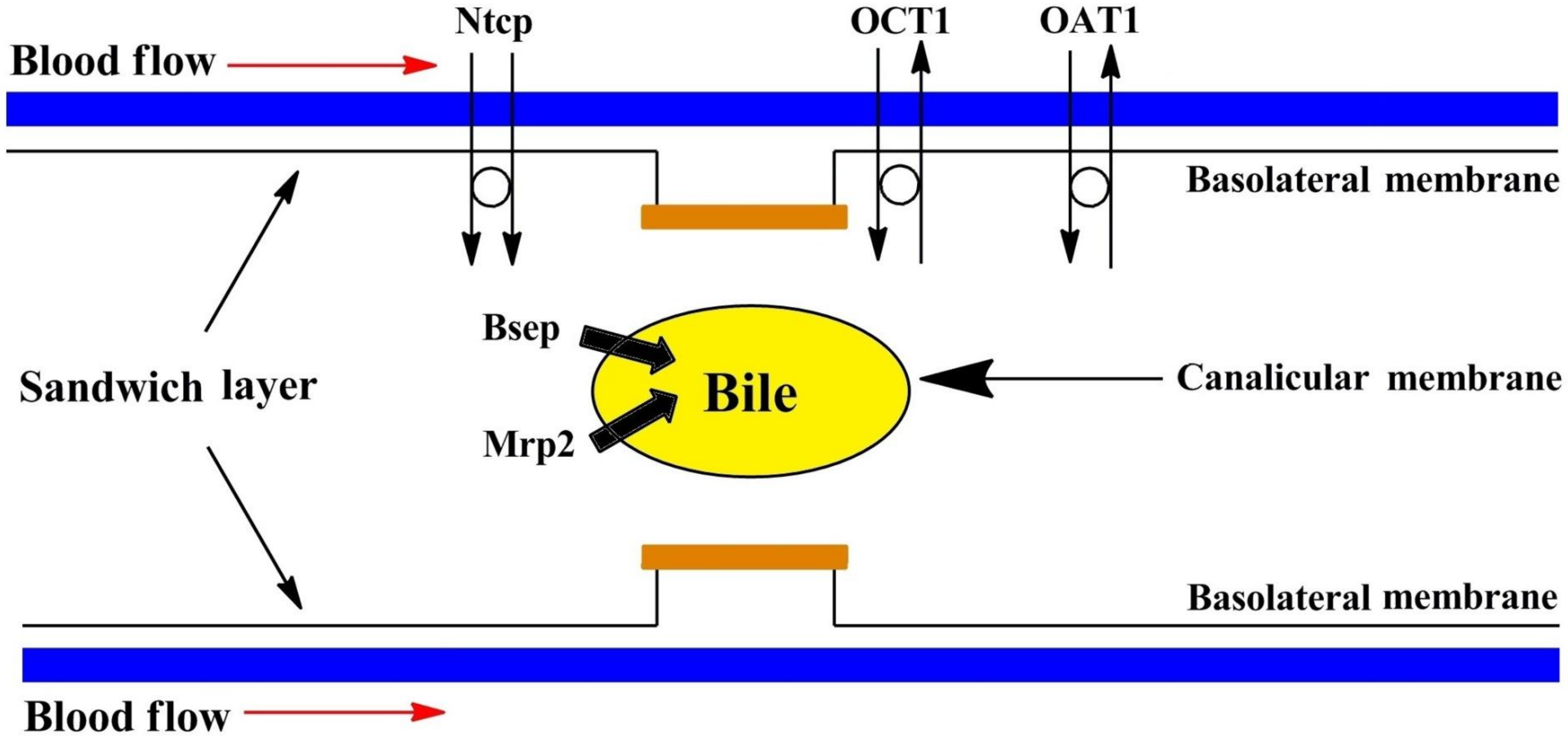

Supplemental Figure 2. Scheme illustrating the polarized expression of transporters in SCHs.

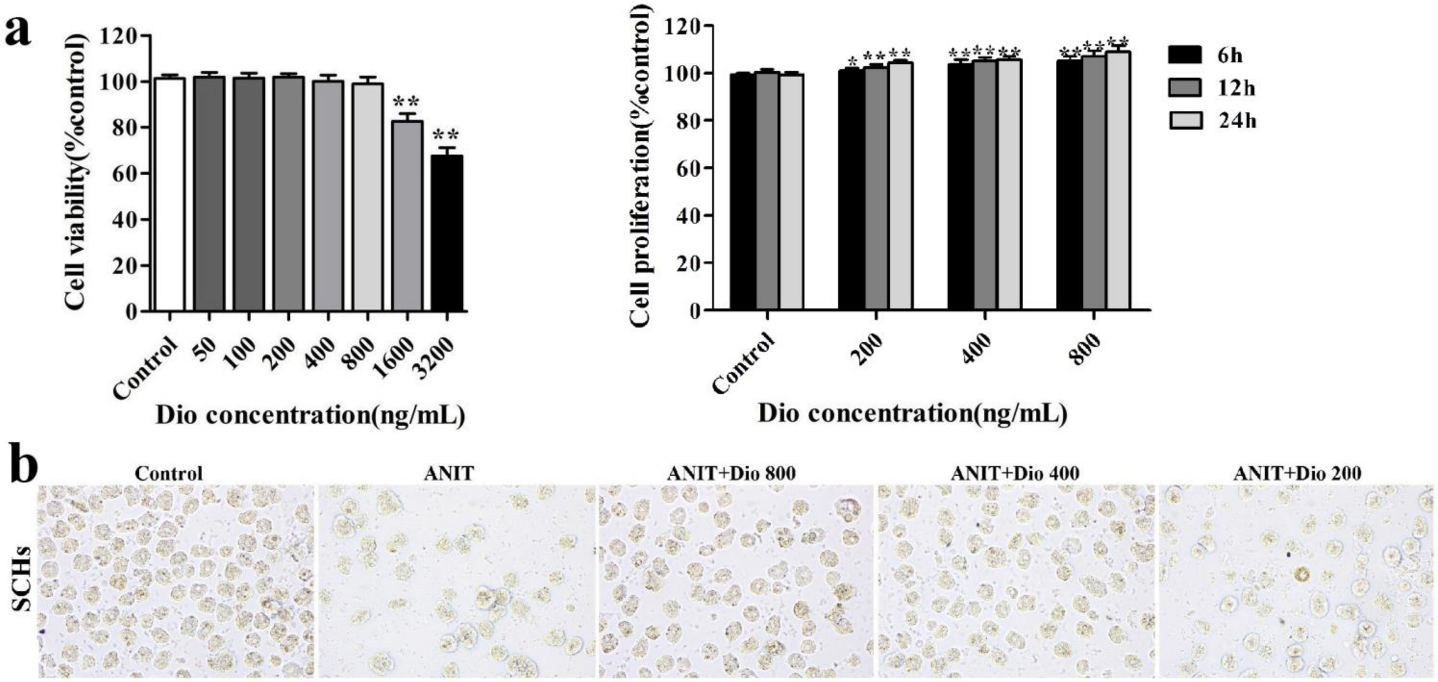

Supplemental Figure 3. Dioscin inhibits proliferation of the primary cultured hepatocytes treated with ANIT. (a) Effects of dioscin on the viability and proliferation of the primary cultured hepatocytes. (b) Effects of dioscin (800, 400 and 200 ng/ml for 24 h pretreatment) on the cellular morphology and structure of SCHs by bright image (× 400 magnification) investigation. Data are presented as the mean ± SD (n = 3). *p < 0.05 and **p < 0.01 compared with control group.

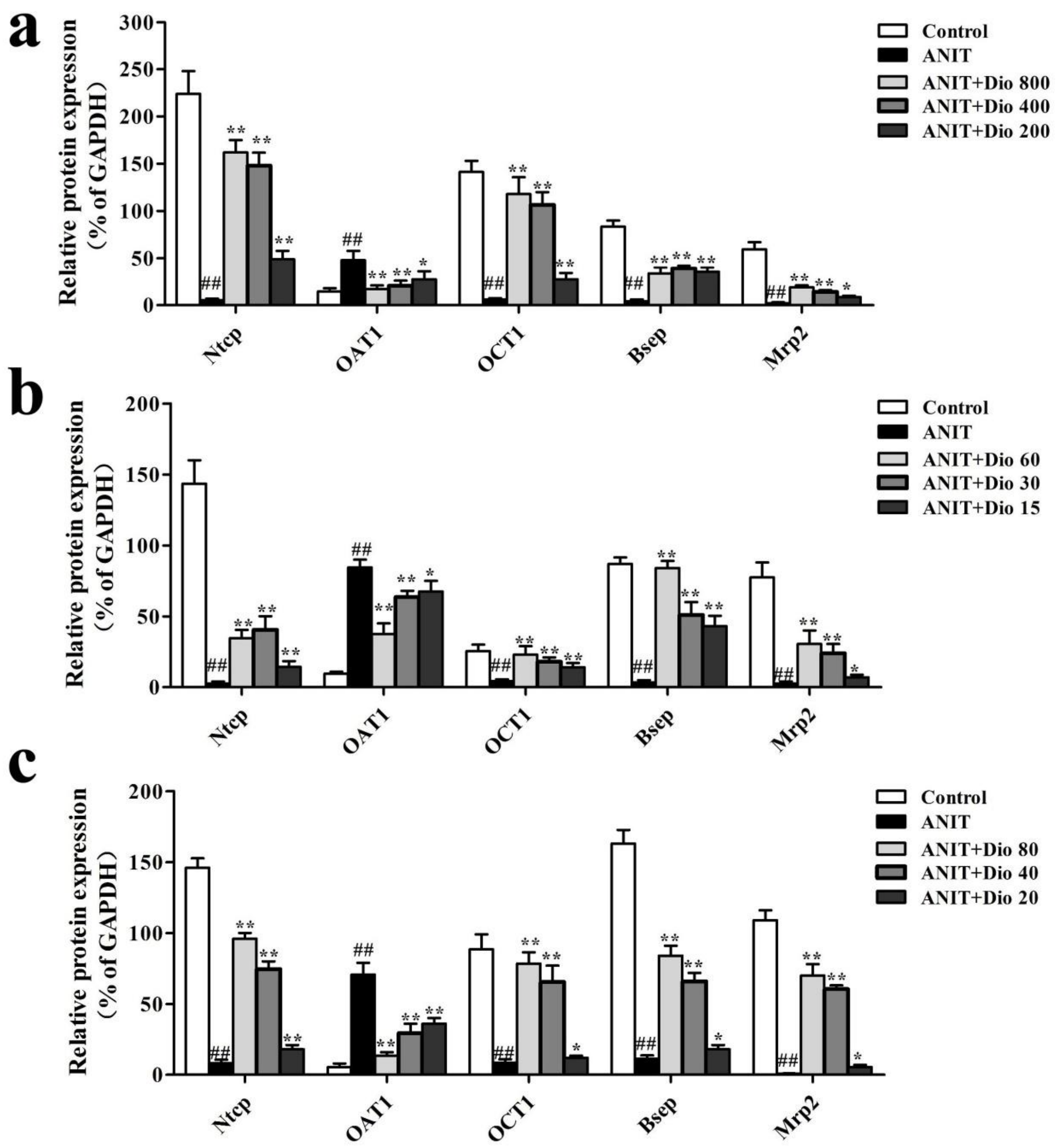

Supplemental Figure 4. Effects of dioscin on the protein levels of Ntcp, OAT1, OCT1, Bsep and Mrp2 in SCHs, rats and mice. Data are presented as the mean ± SD (n = 3). $^{\#\#}p < 0.01$ vs. control groups. $^{*}p < 0.05$ and $^{**}p < 0.01$ vs. model groups.

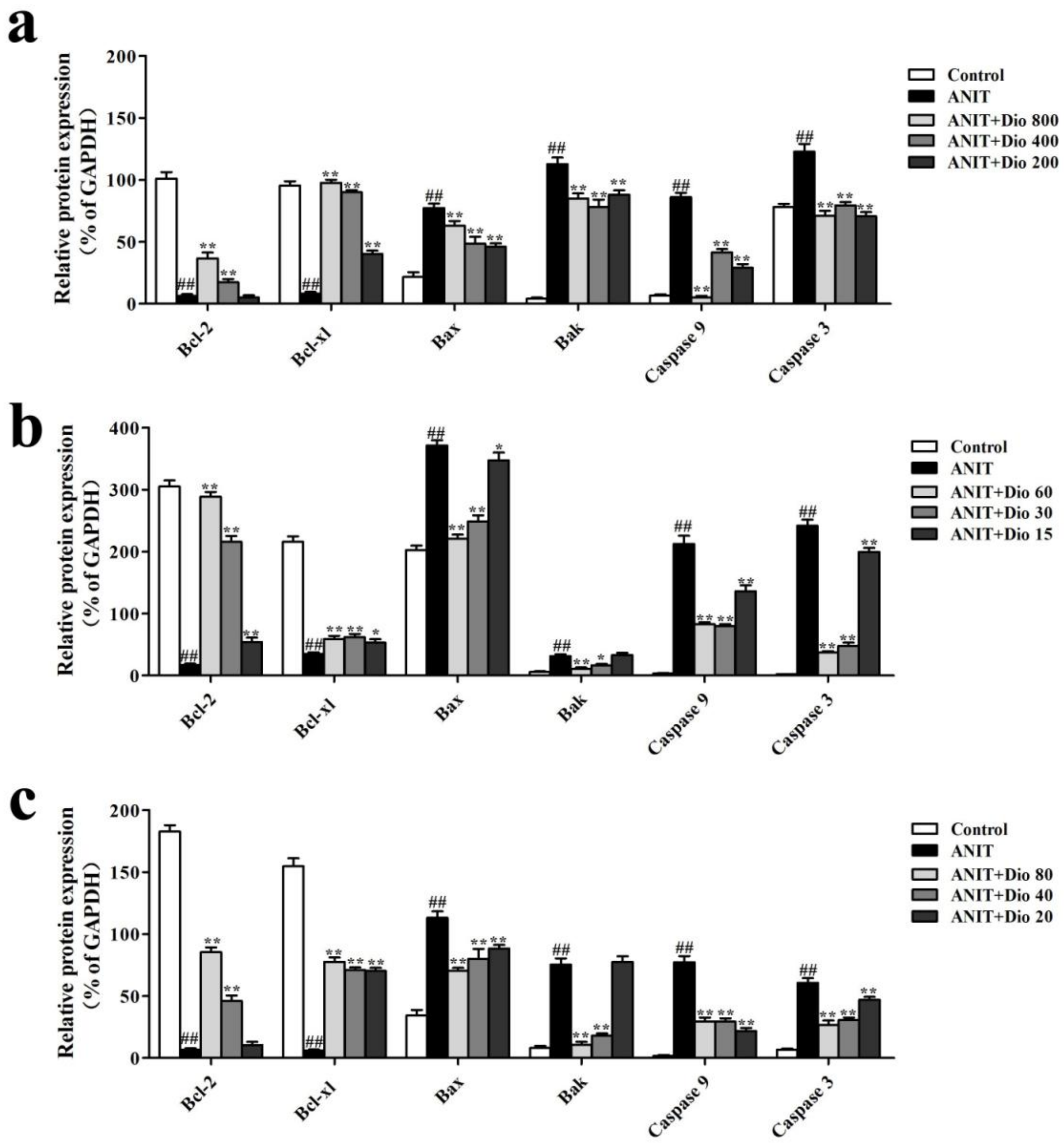

Supplemental Figure 5. Effects of dioscin on the protein levels of Bcl-2, Bcl-xl, Bax, Bak, Caspase 9 and Caspase 3 in SCHs, rats and mice. Data are presented as the mean ± SD (n = 3). $^{\#\#}p < 0.01$ vs. control groups. $^{*}p < 0.05$ and $^{**}p < 0.01$ vs. model groups.

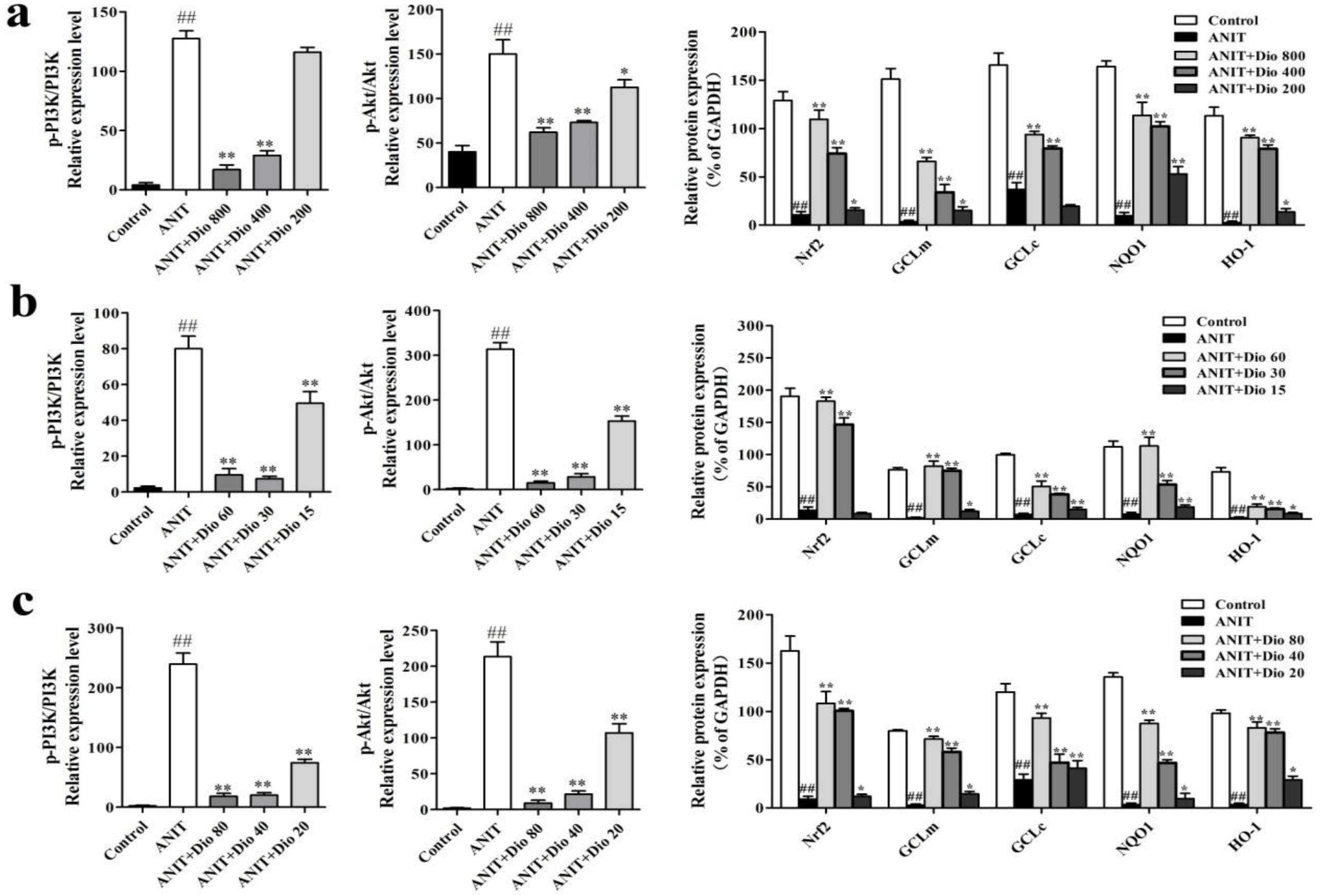

Supplemental Figure 6. Effects of dioscin on the protein levels of pPI3K, p-Akt, Nrf2, GCLm, GCLc, NQO1 and HO-1 in SCHs, rats and mice. Data are presented as the mean ± SD (n = 3). $^{\#\#}p < 0.01$ vs. control groups. $^{*}p < 0.05$ and $^{**}p < 0.01$ vs. model groups.

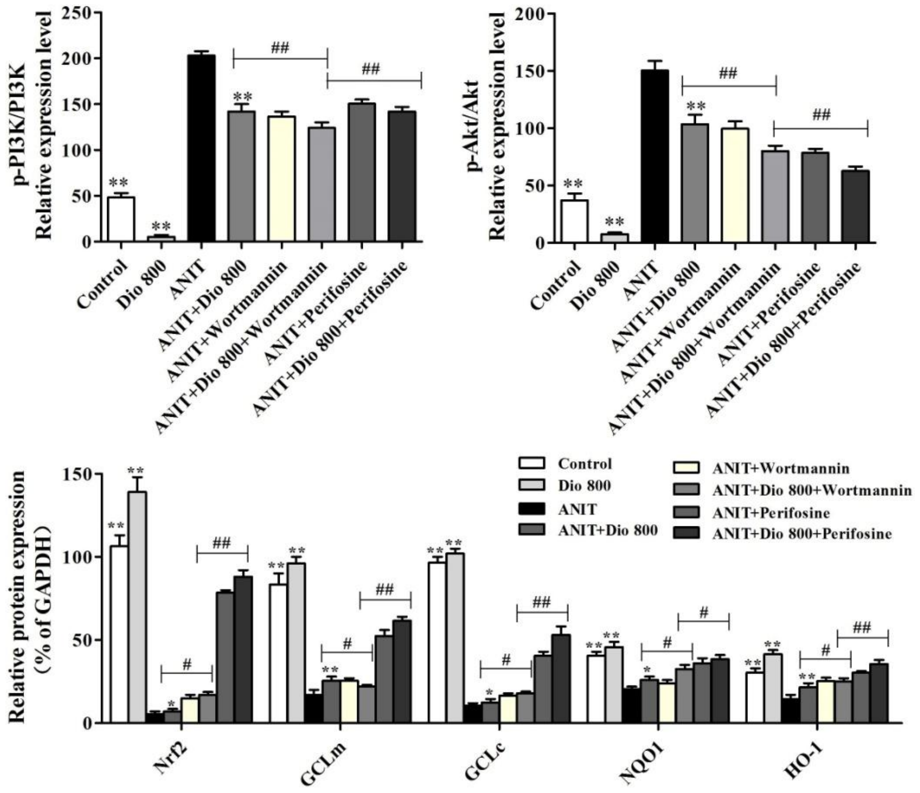

Supplemental Figure 7. Effects of dioscin on the protein levels of p-PI3K, p-Akt, Nrf2, GCLm, GCLc, NQO1 and HO-1 in SCHs treated by wortmannin or perifosine. Values are expressed as the mean ± SD (n = 3). $*p < 0.05$ and $**p < 0.01$ compared with model groups. $^{\#}p < 0.05$ and $^{\#\#}p < 0.01$ compared with ANIT + Dio 800 + wortmannin group.
